# Characterization of Candida spp. from the urinary tract: antifungal susceptibility and virulence traits

**DOI:** 10.1099/jmm.0.002158

**Published:** 2026-05-08

**Authors:** Filipa O. Castro, Francisca Bastos, Ana S. Oliveira, José Martinez de Oliveira, Ana Palmeira de Oliveira, Ana R. Ferrão, Paula G. Pestana, Joana Rolo

**Affiliations:** 1 RISE-Health, University of Beira Interior (UBI), Av. Infante D. Henrique, Covilhã, Portugal; 2 Faculdade das Ciências da Saúde, Universidade da Beira Interior, Covilhã, Portugal; 3 Labfit–Health Products Research and Development Lda, UBImedical, Covilhã, Portugal; 4 Unidade Local de Saúde da Cova da Beira, E.P.E, Covilhã, Portugal

**Keywords:** antifungal susceptibility, *Candida*, epidemiology, urinary infections, virulence factors

## Abstract

**Introduction.** *Candida* spp. are opportunistic pathogens frequently associated with nosocomial infections, and their incidence in urinary tract infections (UTIs) has increased significantly in recent decades, representing a serious public health concern.

**Hypothesis/Gap Statement.** While fungal UTIs are gaining attention, there is a lack of comparative studies linking gender and specific niches to the pathogenic potential and antifungal resistance of *Candida* isolates in the genitourinary tract.

**Aim.** The aim of this study is to characterize *Candida* spp. isolated from the urinary tract by investigating their incidence, antifungal resistance patterns and phenotypic virulence factors – including biofilm formation, germ tube production and adhesion to human epithelial cells – while specifically comparing these characteristics between male and female patients.

**Methodology.** A total of 37 urinary isolates were collected from patients at a Portuguese public hospital between December 2022 and June 2023. Antifungal susceptibility to fluconazole (FLC) and clotrimazole (CLT) was determined via European Committee on Antimicrobial Susceptibility Testing (EUCAST) broth microdilution. Virulence was assessed through crystal violet biofilm biomass quantification, germ tube formation assays for *Candida albicans* and adhesion assays using HeLa cells.

**Results.** *C. albicans* was the most prevalent species, while *Nakaseomyces glabratus* was exclusively found in female patients (30%). All isolates were susceptible to FLC (MIC ≤2 µg ml^−1^) and CLT (MIC ≤1 µg ml^−1^). High biofilm biomass was particularly noted in non-*albicans* species and isolates from hospitalized patients. While isolates from male patients exhibited higher germ tube formation (*P*<0.05), those from female patients demonstrated a potentially greater capacity for adhesion to HeLa cells (*P*<0.05).

**Conclusion.** *Candida* isolates from the urinary tract demonstrate a potential virulence trait that varies by patient gender and clinical setting. The findings suggest that hospitalized female patients and elderly patients harbour isolates with greater pathogenic potential, highlighting the need for continuous epidemiological surveillance to improve UTI diagnosis and treatment.

**Impact Statement**

Urinary tract infections affect millions of people worldwide. Our work reveals that urinary *Candida* isolates are highly virulent, particularly those isolated from hospitalized female patients. Thus, epidemiological surveillance is key to prevent further complications.

## Introduction

Urinary tract infections (UTIs) are the third most common infection in humans after respiratory and gastrointestinal infections, affecting 150 million people each year worldwide [1]. They are also the most common in primary care, affecting one in five adult women in at least one episode during their lifetime [1]. Initially, it was believed that urine was sterile, but it is now widely accepted that the urinary tract of healthy individuals and their urine harbour a vast microbial community. Still, the number of micro-organisms in healthy individuals is generally low, which is enough to consider urine sterile in standard laboratory tests [2,3].

However, when there is an imbalance in the microbiota, a UTI occurs, and it is caused by the presence of pathogens in any part of the urinary system (kidneys, ureters, bladder, urethra), which involves the production, transport, storage and excretion of urine. However, the reproductive organs can also contribute to the microbial burden in this ecological niche, such as the vagina, cervix, penis and genital skin surface [3]. There are various risk factors that contribute to the occurrence of urinary infections, such as age, gender, diabetes mellitus, pregnancy, prolonged hospitalization, urinary obstruction, intensive care unit admission, immunosuppressive therapy, recent use of broad-spectrum antibiotics, previous surgery (urological and nonurological), inadequate hygiene habits, the insertion of foreign objects, urinary tract instrumentation, transplantation, among others [4,5]. There is some evidence indicating that the incidence of UTIs caused by fungal species, especially *Candida* species, has increased by 2–3 times in recent decades and that they are among the leading causes of nosocomial infections, which result in serious public health problems. *Candida* spp. are opportunistic pathogens normally found in the human microbiota, so many people with underlying health conditions or a weakened immune system, such as chronic illnesses, a history of tuberculosis, human immunodeficiency virus infections and cancer are a vulnerable group for nosocomial fungal infections [5,7].

Diagnosing *Candida* species in urine samples presents a significant challenge for physicians, particularly in primary care and infectious diseases units. The difficulty arises from distinguishing between colonization and true infection, as *Candida* is part of the normal microbial flora of the urogenital system and skin in healthy individuals [8,9]. This creates a dilemma for healthcare providers. The presence of *Candida* spp. in the urine can represent a wide range of conditions, from simple sample contamination to more serious issues such as UTIs or even disseminated candidiasis [10,11]. However, the presence of *Candida* in urine does not always indicate an infection requiring treatment.

Furthermore, there are no specific guidelines or standardized protocols for diagnosing *Candida* infections in urine, making it harder to interpret the clinical significance of such findings. The latter can be life-threatening if not recognized and treated appropriately. Although the complications of *Candida* colonization in the urinary tract are associated with a decrease in patients’ quality of life and a significant clinical and economic burden [12], it is crucial to identify *Candida* strains at the species level due to their differences in antifungal and virulence patterns to provide the most effective treatment.

Regarding this, the aim of the present study was to characterize *Candida* isolates obtained from urine, considering the role of predisposing risk factors for carriage of yeast in this ecological niche.

## Methods

### Sample and data collection

Candida strains (*n*=37) isolated from urine were collected, from December 2022 to June 2023, from 35 patients attending the Cova da Beira Hospital Centre, Covilhã, Portugal. The patients attended consultation, emergency or were hospitalized, independently of the reason; in hospitalized patients, samples were obtained directly from indwelling urinary catheters, and for patients attending outpatient consultations or emergency services, urine was collected through midstream clean-catch during spontaneous voiding (uroculture). They were not recruited or selected for this study; the samples constituted convenience samples as they were collected by healthcare professionals for diagnostic purposes – and not specifically for UTIs. The samples were anonymized prior to their inclusion in the study. Patients’ demographic and clinical status, such as age, gender and the presence of known risk factors for the occurrence of UTIs (diabetes mellitus and pregnancy), were recorded for each sample. The samples were divided into clinical groups considering the origin of the culture (hospitalization, consultation, emergency); patients who did not have diabetes or any other known risk factors for UTI were classified as healthy.

### Identification and isolation of *Candida* species

The *Candida* species identification was performed by an automated analysis of the biochemical profiles using Vitek^®^ 2 Compact (BioMérieux, Marcy-l’Étoile, France), regardless of whether they were part of a pure culture or present alongside other micro-organisms, such as bacteria. This approach was adopted because, within the current healthcare framework, there are no standardized guidelines for the diagnosis and treatment of UTIs specifically caused by yeast.

Subsequently, the samples were separately seeded onto Sabouraud Dextrose Agar (SDA; VWR, Radnor, PA, USA) and were duly catalogued according to clinical history by gender and age. In the end, all isolates were stored in cryogenic vials with brain heart infusion (BHI; VWR, Radnor, PA, USA) broth supplemented with 20% glycerol at −80 °C until needed for future analysis.

### Antifungal susceptibility testing

Antimicrobial susceptibility of all the isolates for fluconazole (FLC) (Thermo Fisher Scientific, Waltham, MA, USA) and clotrimazole (CLT) (Sigma-Aldrich, St. Louis, MO, USA) was determined using the broth microdilution test according to the methodological standards detailed by the European Committee on Antimicrobial Susceptibility Testing [13]. While MIC values for FLC were interpreted according to EUCAST (European Committee on Antimicrobial Susceptibility Testing) clinical breakpoints, results for CLT are reported as MICs only, as no clinical breakpoints have been established for this agent.

Firstly, the inocula were prepared by plating the isolates on SDA and incubating for 24 h at 37 °C. Each isolate was suspended in 3 ml of 0.85% NaCl (sodium chloride) to an OD of 0.5 McFarland using a densitometer (Grant-bio, DEN-1, Grant Instruments; Fisher Scientific, Hudson, NH, USA), which corresponds to ∼1×10^6^ c.f.u. ml^−1^. The suspension was diluted twice: first to 1:50 in Roswell Park Memorial Institute (RPMI-1640; Sigma-Aldrich, St. Louis, MO, USA) broth medium, and then to a final dilution of 1:20. The final suspension was further diluted 1:2 in the plate wells. MIC values were determined spectrophotometrically by reading the OD at 600 nm of absorbance (Bio-Rad, Hercules, CA, USA) after 24 h of incubation. Antifungal agents were tested at concentrations ranging from 1 to 32 µg ml^−1^ and were defined as the lowest concentration of antifungal drug that resulted in inhibition of ≥50% of growth in comparison with a drug-free growth control.

### Biofilm biomass quantification

Biofilms were examined by adherence to the bottom of 96-well plates using the crystal violet (CV) staining method. The streaked colonies were grown overnight in Yeast Peptone Dextrose broth (YPD; Fisher Scientific, Hudson, NH, USA) and collected by centrifugation (Hettich Zentrifugen, MiKRO 200R; Sigma-Aldrich, St. Louis, MO, USA) at 13,000 r.p.m. for 2 min, and washed twice with sterile PBS. Cells were prepared at a concentration of 0.5 McFarland (1×10^6^ cells per millilitre) in RPMI-1640 using a densitometer and then transferred (100 µl) to 96-well microplates that were incubated for 24 h at 37 °C. After incubation, the cells were washed three times with sterile PBS; fixed with 100 µl of 99% methanol (Fisher Scientific, Hudson, NH, USA) for 15 min; stained with 100 µl of 0.05% CV (VWR, Avantor, Radnor, PA, USA) solution for 20 min; and resuspended with 150 µl of 33% (v/v) acetic acid (Fisher Scientific, Hudson, NH, USA). The OD readings were taken using a spectrophotometer (Bio-Rad, USA) at an absorbance of 590 nm.

### Germinative tube formation

*Candida albicans* isolates were specifically selected only to evaluate the percentage of germinative tube formation, as this species is known for its ability to produce hyphae under inducing conditions. Yeast cultures were prepared at a concentration of 1 McFarland (2×10^6^ cells per millilitre) in 2 ml sterile PBS and transferred (250 µl) to a suspension with 650 µl of YPD enriched with 10% (v/v) foetal bovine serum (FBS; Biochrom GmbH, Berlin, Germany). The suspension was incubated in an orbital shaker (Argitob 200, Aralab, Sintra, Portugal) at 37 °C for 2 h at 150 r.p.m. After incubation, 10 µl of the culture was collected and observed at an optical microscope (YS100; Nikon, Tokyo, Japan), with 400× magnification, in a Neubauer chamber (Optik-Labor, Görlitz, Germany). The percentage of cells expressing the germinative tube was visually counted out of the total number of cells, and the following formula was used:


$$\%\;of\;cells\;with\;germ\;tube=\frac{Number\;of\;germ\;tube\;cells}{Total\;number\;of\;cells}\times 100$$

### HeLa cells infection with *C. albicans* strains

For this procedure, five representative *C. albicans* samples (three isolates from urine samples of male patients and two isolates from urine samples of female patients) were selected to test adhesion to a human cell line derived from the cervical epithelium with adenocarcinoma (HeLa cells, ATCC^®^ CCL-2™) [14]. The cells were cultured as monolayers in T-flasks in Dulbecco’s Modified Eagle’s Medium (DMEM; Sigma-Aldrich, St. Louis, MO, USA) supplemented with 10% inactivated FBS (Gibco, Thermo Fisher Scientific, Hudson, NH, USA), 1.5 g l^−1^ sodium bicarbonate (Gibco, Thermo Fisher Scientific, Hudson, NH, USA) and a mixture of 100 U ml^−1^ penicillin and 100 µg ml^−1^ streptomycin (Sigma-Aldrich, St. Louis, MO, USA). Cells were cultured for 24–48 h at 37 °C in a 5% CO_2_ (carbon dioxide) atmosphere until fully differentiated. The used media were changed every 2–3 days. After reaching confluence, the cells were subcultured every week, following the supplier’s recommendations. The HeLa cells (2×10^5^ cells per well) were seeded in six-well culture plates and were incubated for 24 h at 37 °C until confluence. Then, *C. albicans* cells were suspended in DMEM medium, supplemented with 2% FBS at 0.5 McFarland (1×10^6^ cells per millilitre per well) and used to infect the cells for 2 h at 37 °C to allow adhesion. After incubation, the cells were washed twice with 1 ml of 1× PBS to remove any suspended yeasts. Yeasts adhering to the HeLa cells were detached by adding 1 ml of 0.01% TrypLE^TM^ Express Enzyme solution; then 10 µl of the solution was transferred to the respective wells in a new sterile 96-well plate with 90 µl of sterile PBS in quadruplicate. Serial dilutions were performed (1:10 up to a 10⁻⁴ dilution), and 5 µl of each dilution was transferred to SDA plates in duplicate. The plates were incubated at 37 °C for 24 h, followed by colony counting from the SDA plates.

### Ethics statement

The study was registered and approved by the Ethics Research Committee of Cova da Beira Hospital Centre (Unidade Local de Saúde Cova da Beira, study 81/2023). All samples were assigned a numerical code at the time of sample collection and processed anonymously.

### Statistical analysis

The Student’s t-test was used to compare demographic and epidemiological data regarding the population under study between male and female patients. The results for antifungal susceptibility of each isolate were expressed as the average of two independent assays with two replicates; for biofilm biomass quantifications, results were expressed as the average of two independent assays with four replicates; for germinative tube formation of each *C. albicans* isolate, results were only validated when there were two tests with a difference of less than 15% in germ tube formation. This analysis was performed using GraphPad Prism 8 software and applying the variance test (one-way ANOVA). Furthermore, the statistical significance was admitted for *P*-values <0.05.

## Results

### Population characteristics

**Fig. 1.**
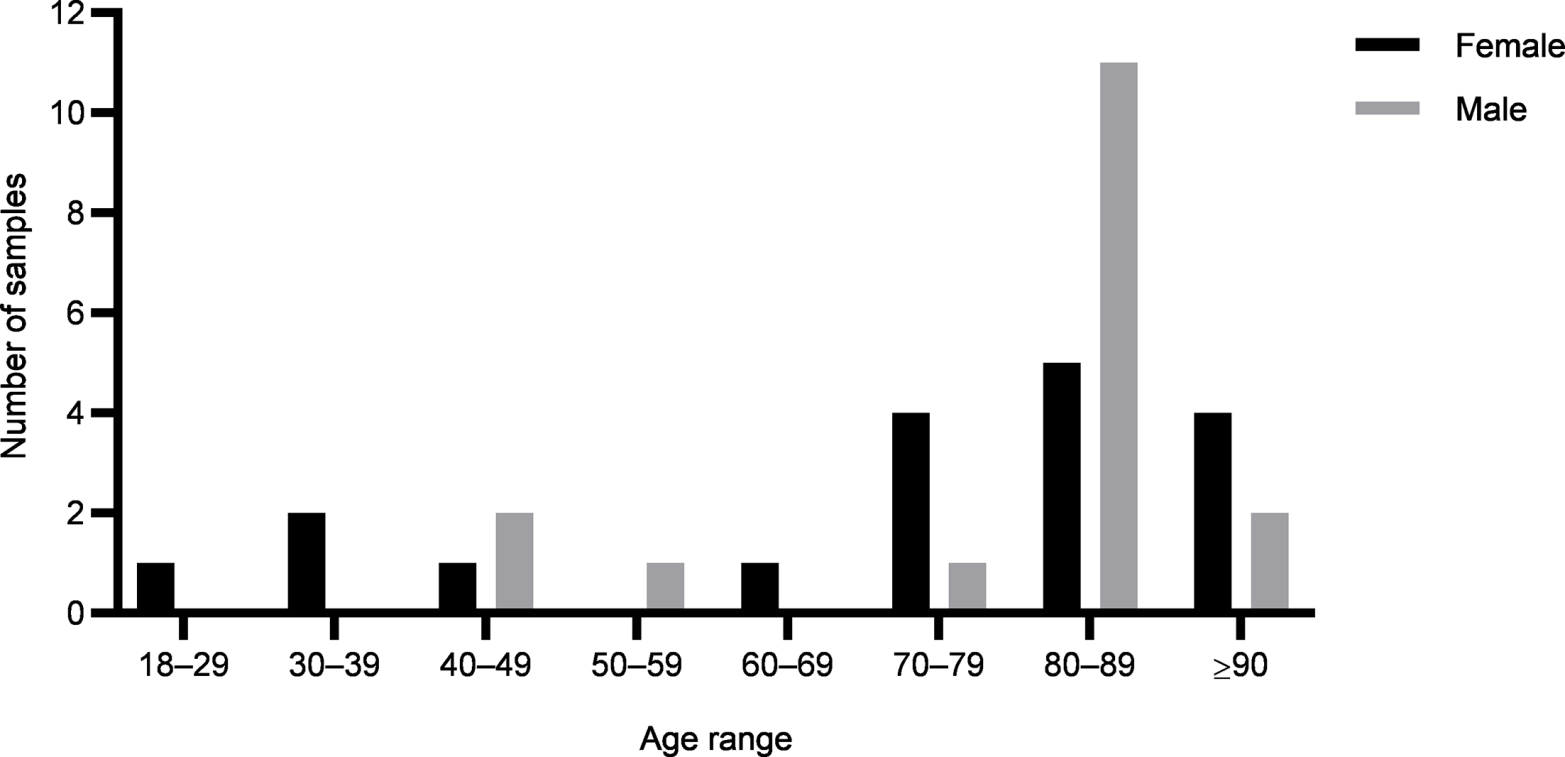
Distribution of urine samples positive for *Candida* spp. by age range and patient sex. Black bars represent female patients, and grey bars represent male patients.

During the study period, *Candida* spp. were detected in the urine of 35 adult patients; 18 (54%) patients were female, and 17 (46%) patients were male. Among female patients, two provided two separate samples each; all male patients contributed a single sample (Table 1). The female patients’ ages ranged from 28 to 95 years with a median of 72 years; the highest number of isolates collected was recorded between the ages of 80 and 89 (*n*=5); four isolates were obtained from patients aged 70–79 years and another four from patients aged 90 years or older. The male patient’s ages ranged from 41 to 90 years with a median of 78 years; eleven (65%) patients were aged between 80 and 89 years; two (12%) patients were between 40 and 49 years; and two were older than 90 years (Fig. 1). Regarding the hospital setting, 22 patients (63%) were hospitalized, 8 (23%) attended emergency medical care, and 5 (14%) attended internal medical clinical wards (Table 1). Although most patients contributed a single sample, the study included two specific clinical cases (both female patients) where two isolates were collected from the same patient on different days. These cases involved one emergency department patient and one hospitalized patient, both of whom presented persistent clinical symptoms following the completion of treatment. These isolates were analysed independently for the ability to express virulence factors and antifungal susceptibility, given the clinical relevance of investigating persistent strains.

**Table 1.** Frequency of urinary colonization with *Candida* spp. among patients according to their socio-demographic features

	*C. albicans*	*C. parapsilosis*	*C. guilliermondii*	*C. ciferrii*	*N. glabratus*
**Gender**					
Female	13	1	–	–	6*
Male	14	1	1	1	–
**Age range**					
18–29	–	1	–	–	–
30–39	2	–	–	–	–
40–49	2	–	–	–	2
50–59	–	–	1	–	–
60–69	1	–	–	–	–
70–79	5	–	–	–	1
80–89	14	1	–	1	–
≥90	4	–	–	–	2
**Samples origin**					
Emergency	5	–	1	–	3
Consults	3	1	–	1	–
Hospitalized	19	1	–	–	3

* There are two patients who were admitted on different days, and for each day, a urine sample was collected.

- No isolates of this species have been collected (n=0).

### Risk factors

The frequency of *Candida* spp. colonization among participants with various potential risk factors for UTIs was evaluated by comparing the group classified as healthy (with no known underlying health conditions) with the group with existing health issues (including diabetes mellitus, immunosuppressive treatment, pregnancy and invasive procedures). The frequency of colonization in the group with existing health issues was higher in a hospital environment (*n*=11) compared with those not hospitalized (*n*=8), as shown in Table 2.

**Table 2.** Incidence of urinary colonization of *Candida* spp. among patients with potential risk factors

Potential risk	**Hospitalized**	Emergency/outpatients
Diabetes mellitus	10	3
Immunosuppressive treatment	–	2
Pregnancy	–	3
Invasive procedures*	1	–

* ICU (Intensive Care Unit) patients with indwelling catheters or who were exposed to broad-spectrum antimicrobials.

Among host-related factors, the highest frequency of urinary colonization by *Candida* spp. was observed in patients with diabetes mellitus (Table 2). In contrast, the frequency of *Candida* colonization was lower among the immunosuppressed group.

### *Candida* species distribution

**Fig. 2.**
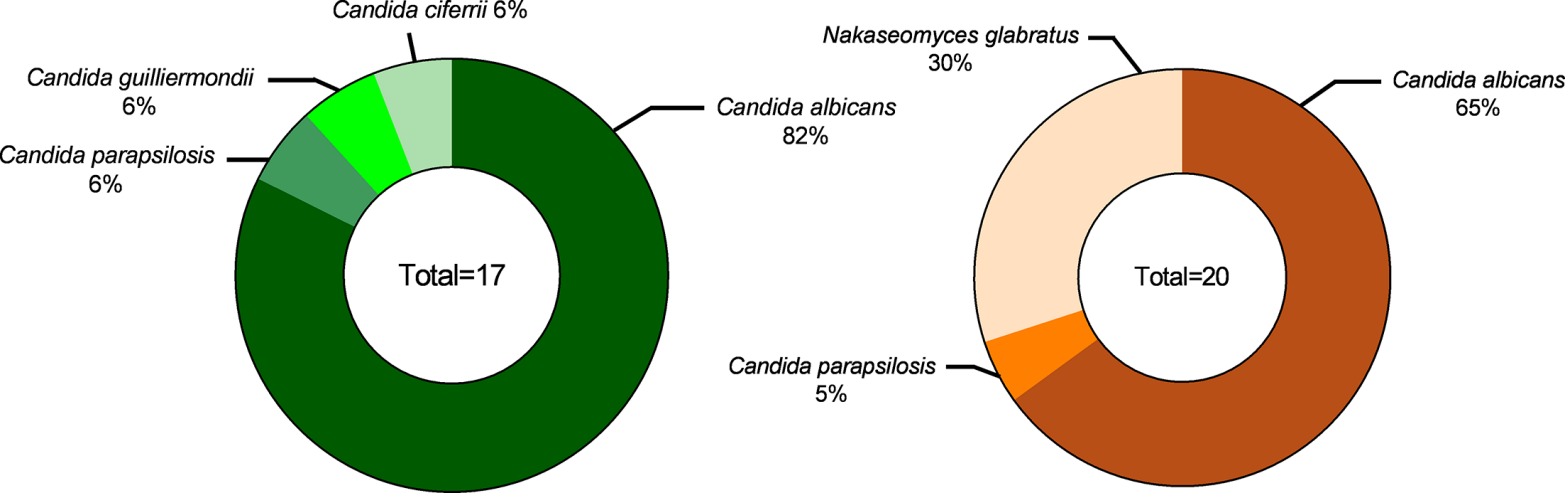
Distribution of *Candida* spp. isolates: (a) from male patients (*n*=17) and (b) from female patients (*n*=20).

The distribution of urinary cultures by species was analysed through automated biochemical profiling using the Vitek^®^ 2 compact system. In this analysis, *C. albicans* emerged as the most frequently isolated yeast (Fig. 2) . A notable finding was the exclusive presence of *Nakaseomyces glabratus* in female urinary samples, where it accounted for 30% of the isolates (Fig. 2). Furthermore, *C. albicans* showed a higher association with male patients (82%) compared with female patients (65%) with urinary infections. Among male patients, three non-*albicans Candida* species were identified: *Candida parapsilosis* (6%), *Candida guilliermondii* (6%) and *Candida ciferrii* (6%). In contrast, only two non-*albicans Candida* species, *C. parapsilosis* (5%) and *N. glabratus* (30%), were found in female patients.

### Antifungal susceptibility

Antimicrobial susceptibility testing was performed for FLC and CLT using the broth microdilution method to determine the MIC values. The results, represented by the MIC50 (concentration needed to inhibit 50% of microbial growth), indicated that all species tested exhibited low MIC values for both antifungals (Table 3). For FLC, the MIC for all isolates was ≤2 µg ml^−1^, indicating that the yeasts were susceptible to this antifungal (for *N. glabratus*, this MIC is indicative of susceptibility with increased exposure). For CLT, the MIC for all isolates was ≤1 µg ml^−1^, also indicating susceptibility to CLT.

**Table 3.** Comparison of the MIC of FLC and CLT (μg ml^−1^) for *C. albicans* and non-*albicans Candida* spp. isolated from male and female patients

Species	Agent	Female	Male
*C. albicans* (*n*=27)	FLC	≤2	≤2
	CLT	≤1	≤1
*C. parapsilosis* (*n*=2)	FLC	≤2	≤2
	CLT	≤1	≤1
*C. guilliermondii* (*n*=1)	FLC	–	≤2
	CLT	–	≤1
*C. ciferrii* (*n*=1)	FLC	–	≤2
	CLT	–	≤1
*N. glabratus* (*n*=6)	FLC	≤2	–
	CLT	≤1	–

As predicted, yeasts demonstrated greater sensitivity to CLT when compared with FLC, suggesting that CLT may be a more effective antifungal agent against these yeast isolates in this study (Table 3). In summary, all isolates were susceptible with increased exposure to both antifungals, but CLT showed superior activity against the yeasts tested in comparison with FLC.

### Biofilm biomass

**Fig. 3.**
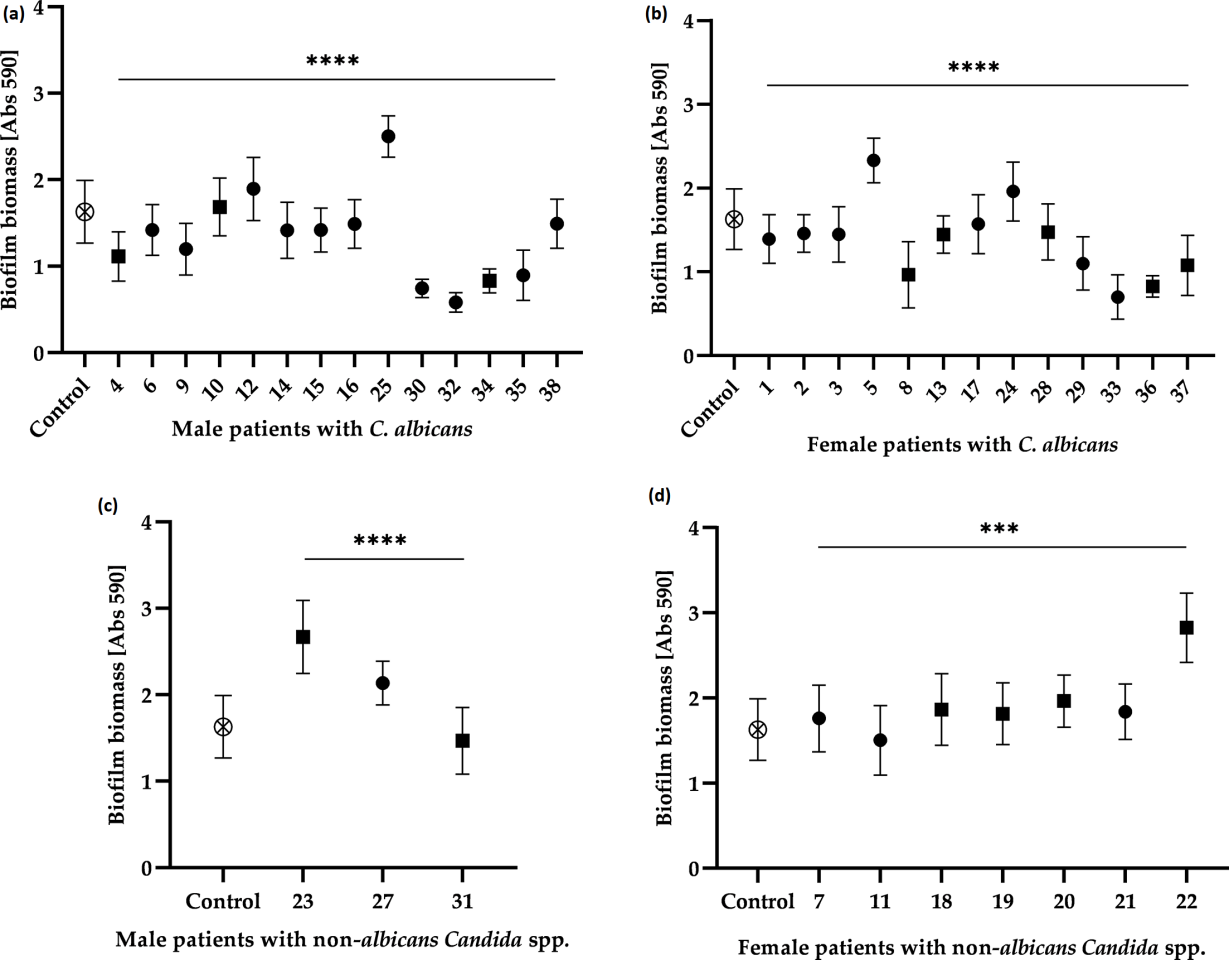
Absorbance values of CV obtained from biofilms formed after 24 h by isolates of *C. albicans*: (a) from male patients; (b) from female patients; and non-*albicans Candida* isolates: (c) from male patients; (d) from female patients. ⊗ indicates the control, which refers to the *C. albicans* reference strain ATCC 10231; *y*-axis represents the OD at 590 nm, reflecting biofilm biomass; *x*-axis represents individual *Candida* isolates; ● indicates isolates from hospitalized patients; ■ indicates isolates from emergency care/outpatients services. **** represents an overall statistically significant difference (*P*<0.0001), ***P<0.001, as determined by the Kruskal–Wallis test.

The quantification of biofilm biomass was carried out using CV staining after 24 h of biofilm formation on polystyrene plates for all 37 *Candida* species collected; the correlation and significance values obtained are shown in Fig. 3. All isolates were capable of producing biofilms, with only small differences in biofilm production between isolates collected from male and female patients. While biofilm production was similar, biofilms produced by non-*albicans Candida* species exhibited higher biomass production compared with those produced by *C. albicans*, regardless of the source of isolation (Fig. 3c, d).

Upon examining the biofilm biomass profile values, it was observed that, in general, isolates obtained from hospitalized patients demonstrated a higher biofilm production capacity compared with isolates obtained from patients attending emergency care/outpatient services. However, significant biofilm biomass was also observed in *C. guilliermondii* and *C. parapsilosis* (isolates 23 and 22, respectively), which are associated with these patients. This suggests that certain community-acquired *Candida* species can also exhibit robust biofilm production, potentially influencing infection outcomes.

### Germinative tube

*C. albicans* has the capacity to exhibit either the typical yeast cell form, reproducing by budding, or to form germ tubes extending from the yeast cells. The ability to form germ tubes is an important virulence factor for *C. albicans*, especially in mucosal and systemic infections. Other yeast species identified in this study, such as *N. glabratus*, are unable to form true hyphae under standard inducing conditions and were therefore excluded from the hypha formation assay [15]. In this study, 27 isolates were selected to determine germ tube formation, with 14 isolates from male patients and 13 from female patients.

**Fig. 4.**
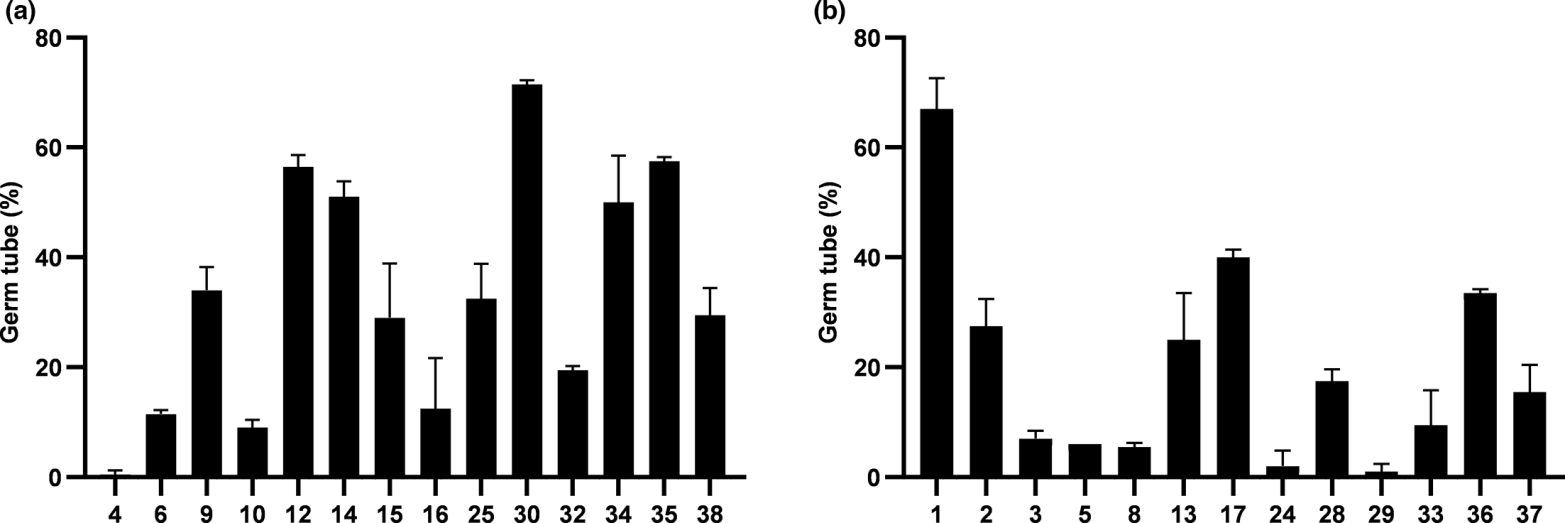
Percentage of germ tube production (%) after 2 h of incubation from all samples with *C. albicans*, according to: (a) male patients and (b) female patients. (*) indicate samples obtained from diabetic patients.

The graphical analysis in Fig. 4 reveals significant differences between the groups of isolates from male and female patients, with higher percentages of germ tube formation observed in the male group overall. However, the average percentage of germ tube formation varied depending on the clinical conditions of the patients. Yeasts isolated from diabetic patients demonstrated greater germ tube production (>50%) compared with those from patients without diabetes, although the latter group could have other unknown conditions that may influence germ tube formation. This suggests that diabetes may enhance the ability of *C. albicans* to form germ tubes, potentially contributing to its virulence in diabetic individuals.

### Adhesion to HeLa cells

**Fig. 5.**
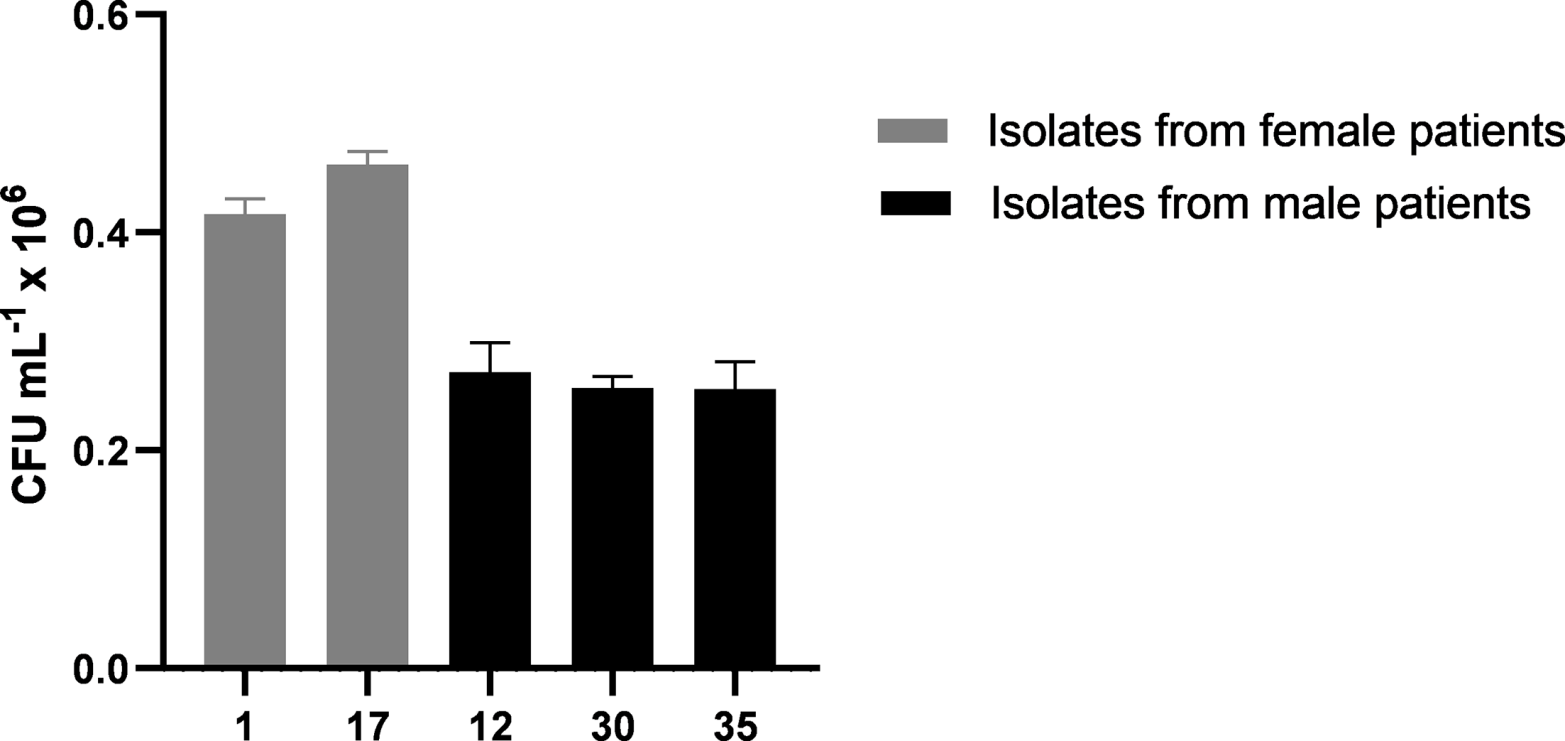
The adhesion capacity of *C. albicans* strains to HeLa cells was assessed after a 2 h incubation period with individual strain isolates from female patients (1 and 17) and male patients (12, 30 and 35). A statistically significant difference (**P<0.05) was observed between the two groups (female vs. male isolates), determined by the Mann–Whitney test. CFU: colony forming units.

The adhesion and colonization of HeLa cells by *C. albicans* were characterized by the recovery of c.f.u. ml^−1^ after 2 h of infection. For this, five isolates were selected based on patient sex and germ tube formation capacity. Specifically, we chose the two female- and three male-derived strains showing the highest germ tube formation percentages to assess potential sex-related differences in adhesion behaviour. The samples 12, 30 and 35 were from urine cultures of male patients; 1 and 17 were from urine cultures of female patients. The percentage of the suspension that adhered to the HeLa cells was determined, and the number of c.f.u. ml^−1^ recovered was quantified, as shown in Fig. 5. The graph shows that both isolates from urine cultures of female patients (1 and 17) exhibited a higher capacity for adhesion/infection. Therefore, the adhesion to HeLa cells was weaker in the yeasts isolated from urine cultures of male patients.

## Discussion

In recent years, the prevalence of both mucocutaneous and systemic infections caused by *Candida* species has increased, particularly among patients with chronic illnesses or compromised immune systems. While *Candida* infections represent a significant public health concern globally, their epidemiological characterization in the genitourinary tract remains challenging due to the lack of comparative studies linking gender and niche. Although UTIs caused by fungi have gained increased attention in research, the role of this microbial community in human health is still not fully understood. Nonetheless, there has been a surge in reports linking resistance to all available antifungal agents [16,19]. These findings are clinically relevant in the context of candiduria, where resistance to commonly used antifungals such as FLC has been documented in both *C. albicans* and non-*albicans* species. This rising resistance may compromise treatment effectiveness and contribute to persistent infections and higher recurrence rates. It thus provides a comprehensive overview of the microbiological landscape, shedding light on the profile of *Candida* spp. in urine and its influence within this healthcare institution.

The sociodemographic characterization of the study population reveals that gender and age are crucial risk factors for the isolation of *Candida* spp. Among the total urinary tract isolates, 54% were from female patients aged between 28 and 95 years, while the remaining isolates were from male patients aged between 41 and 90 years. These data are consistent with the literature, as studies show a higher prevalence of *Candida* in urine among women and older patients [19,21]. Among these risk factors, the correlation with the risk of candidemia in patients over 70 years old is particularly concerning, as most patients in this study were hospitalized. These underlying conditions may predispose them to develop invasive candidiasis, which is associated with high global mortality rates [22]. Through the analysis of the origin of the urine samples, the highest incidence of *Candida* in urine samples occurred in hospitalized patients (62%), followed by those in the emergency department (24%) and, lastly, those attending outpatient consultations (14%). These findings are consistent with the retrospective study by Lima *et al.* [23], which examined 106 patients diagnosed with candiduria. Among these, 31% were hospitalized in the intensive care unit and 31% in the emergency department [23]. Other risk factors that were studied by us included diabetes mellitus (37%), pregnancy (9%) and immunosuppressive treatments (6%). Diabetes is a well-established risk factor for the development of nosocomial UTIs [4,24, 25]; pregnancy increases the risk of *Candida* infections due to hormonal changes that alter the vaginal environment, making it more favourable for fungal growth. Additionally, immune system adaptations during pregnancy may reduce the body’s ability to control infections, and the pressure from the growing uterus can cause urinary stasis, raising the likelihood of *Candida* colonization in the urinary tract [26,27].

*C. albicans* was the most prevalent species in both male and female patients. However, in female patients, the second most frequently isolated species was *N. glabratus* (30%), followed by *C. parapsilosis* (5%). In males, *N. glabratus* was not present, but * C. parapsilosis*, *C. guilliermondii* and *C. ciferrii* were found at equal prevalence. However, there is limited evidence in the literature regarding the distribution of *Candida* spp. in the male urinary tract due to the lower incidence of candiduria compared with females.

The characterization of susceptibility profiles is essential for the effective treatment of infections and for controlling antimicrobial resistance. Azole agents, such as FLC, are triazole antifungals with fungistatic, rather than fungicidal, activity [28], while CLT is an imidazole antifungal with properties mediated by its interaction with ergosterol synthesis. This interaction increases the permeability of the yeast cell wall, disrupting its structure and function [29]. FLC is the antifungal agent most used in clinical practice in Portugal, followed by CLT, which is mostly used by the community to treat superficial infections [30]. In this study, all yeasts were found to be either susceptible or susceptible with increased exposure to both antifungals, indicating that these compounds are still useful to treat these yeast infections. In the vast majority of studies conducted worldwide, it has been demonstrated that *Candida* species isolated from urine show sensitivity to antifungal agents, which is in accordance with our study, but exhibit a high capacity for infection, likely due to their ability to form biofilms, adhere to uroepithelial cells and resist host immune responses [31].

We also evaluated the virulence factors associated with nosocomial infections by *Candida* species, which have been linked to the use of medical devices, such as catheters, due to the formation of biofilms that, in turn, increase the risk of pathogenicity and antimicrobial resistance. Therefore, biofilms have been correlated with the growth of opportunistic micro-organisms, such as *Candida* [31]. In this study, larger quantities of biofilm biomass were observed in *C. parapsilosis*, with this isolation being associated with a pregnant patient. The isolates of *N. glabratus* exhibited similar biomass values, while among the *C. albicans* yeasts, the isolate number 5 showed the highest total biomass, being associated with an 87-year-old diabetic patient who was hospitalized. However, these results do not align with previous studies, which report *C. albicans* as a strong biofilm producer, followed by *N. glabratus* and, lastly, *C. parapsilosis* [32]. Among the isolates from urine cultures of male patients, *C. guilliermondii* showed the highest biofilm biomass, followed by *C. albicans* and *C. parapsilosis*, all of which were associated with hospitalized patients. Nonetheless, as this comparison is based on a single isolate, conclusions regarding its relative biofilm-forming ability should be interpreted with caution.

Considering other virulence factors, many studies have reported that *C. albicans* can exist in two phases: the yeast phase and the filamentous hyphal phase, with the hyphal form being associated with invasive infection, capable of actively penetrating epithelial cells and stabilizing mature biofilms [33,34]. Conversely, the yeast phase is responsible for the distribution and dissemination of *C. albicans*. Therefore, the transition between the yeast and hyphal phases is crucial for the progression and regulation of infection processes [35]. In this study, the evaluation of germ tube formation involved quantifying hyphae in each isolate. It was found that 36% of the 14 isolates from male urinary cultures and 8% of the 13 isolates from female urinary cultures showed a significant number of hyphae formed (≥50%). Among the male urinary culture samples, isolate 30 exhibited the highest percentage of hyphae formed, ∼71%, while isolate 4 showed the lowest percentage, with just 1%. Furthermore, the risk factors associated with the isolates are relatively similar; of the five isolates, four were from patients aged between 80 and 90 years who were hospitalized. Three of these patients were diagnosed with candiduria and had diabetes. However, there are no conflicting data in the literature regarding these results, indicating that further investigation is required in this area of study, particularly concerning urinary infections. In contrast, in the female urinary culture samples, isolate 1, with 67% hyphal formation, was from a diabetic woman hospitalized in the ICU.

Lastly, the adhesion of *C. albicans* to HeLa cells was greater in isolates from urinary cultures of female patients. Therefore, isolates from the male urinary tract demonstrated significantly lower adhesion compared with the previous isolates, as would be expected, because of differences in anatomy and the hormonal environment, both of which may create conditions that support fungal colonization. Thus, *C. albicans* isolated from the female urinary tract exhibited a significantly increased capacity for invasion and yeast–host interactions.

Despite the promising results, this study is subject to several limitations that must be considered when interpreting the data. Firstly, the sample size (*n*=37) is relatively small and was sourced from a single healthcare institution, which may restrict the generalizability of the findings to other geographical regions or clinical contexts. Furthermore, while *in vitro* cellular adhesion assays provide significant indicators of pathogenicity, these laboratory results should be interpreted with caution, as they may not fully capture the complexity of host–pathogen interactions in a clinical environment. Future research involving a larger patient cohort and longitudinal clinical data is necessary to validate these correlations and their impact on therapeutic success.

## Conclusions

Our results demonstrated that the clinically important species in women include *C. albicans* and *N. glabratus*. In men, *C. albicans* is the predominant species, which, in turn, represents a high risk, particularly in hospital settings, where they emerge as nosocomial pathogens in chronically ill and/or post-operative patients. Therefore, the distribution of yeast species in the female urinary tract differs from that in the male urinary tract, although the antifungal susceptibility profiles are the same. The *Candida* isolates from the urinary tract exhibited relevant virulence factors, highlighting the need for further studies to unravel the pathogenic potential of urinary yeast. Nonetheless, the yeasts obtained from urinary cultures of female patients suggest a higher capacity for infection and virulence compared with male patients’ isolates. Furthermore, this study found that the isolates with the highest virulence were from female hospitalized patients of advanced age or who were pregnant, representing a significant risk group due to *Candida*’s ability to adhere to the inanimate surfaces of medical devices inserted into patients. Elucidating the key mechanisms in urinary infections is crucial for advancing the understanding and treatment of invasive *Candida* infections.

## Abbreviations

BHI: brain heart infusion
CLT: clotrimazole
CV: crystal violet
DMEM: Dulbecco’s Modified Eagle’s Medium
EUCAST: European Committee on Antimicrobial Susceptibility Testing
FBS: foetal bovine serum
FLC: fluconazole
RPMI: Roswell Park Memorial Institute
SDA: Sabouraud dextrose agar
UTIs: urinary tract infections
YPD: yeast peptone dextrose broth
